# The Electric Field Morphology of Plasmonic Picocavities

**DOI:** 10.1021/acs.nanolett.5c01999

**Published:** 2025-06-25

**Authors:** Tommaso Giovannini, Luca Nicoli, Stefano Corni, Chiara Cappelli

**Affiliations:** † Department of Physics and INFN, 9318 University of Rome Tor Vergata, Via della Ricerca Scientifica 1, 00133 Rome, Italy; ‡ Scuola Normale Superiore, Piazza dei Cavalieri 7, 56126 Pisa, Italy; ¶ Department of Chemical Sciences, University of Padova, via Marzolo 1, 35131 Padova, Italy; § CNR Institute of Nanoscience, via Campi 213/A, 41125 Modena, Italy; ∥ IMT School for Advanced Studies Lucca, Piazza San Francesco 19, Lucca 55100, Italy

**Keywords:** picocavity, nanocavity, atomistic, modeling, adatom, SERS

## Abstract

Picocavities are plasmonic nanostructures featuring atomistic defects within subnanometer gaps. Such a unique morphology enables extreme light confinement at subnanometer scales and drives substantial field enhancements with applications from molecular sensing to plasmon-driven catalysis. However, the impact of atomistic defects on the plasmonic field morphology, which ultimately determines light–matter interactions at the nanoscale, remains largely unexplored due to the limitations of traditional theoretical models. Here, we employ the frequency-dependent fluctuating charges and dipoles (ωFQFμ) approach, an atomistic yet computationally efficient method previously validated against time-dependent density functional theory calculations, to reveal the plasmonic field morphology in gold picocavities composed of thousands of atoms. Our results uncover pronounced field inhomogeneities induced by the atomic-scale defects, which may trigger novel effects where electric field gradients are pivotal. Our findings establish the physical foundations for rationalizing experimental observations and guiding the design of next-generation nanophotonic devices with unprecedented control over atomic-scale field confinement.

## Graphical abstract

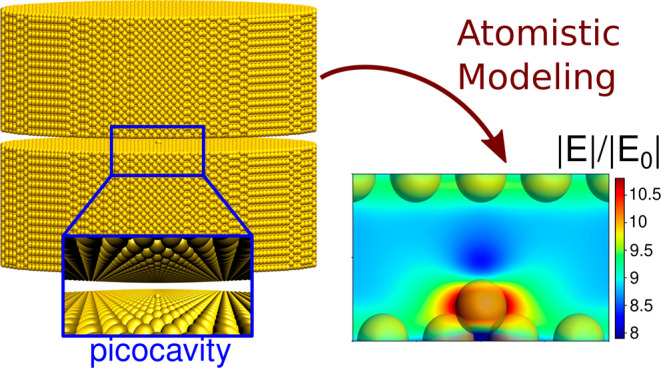

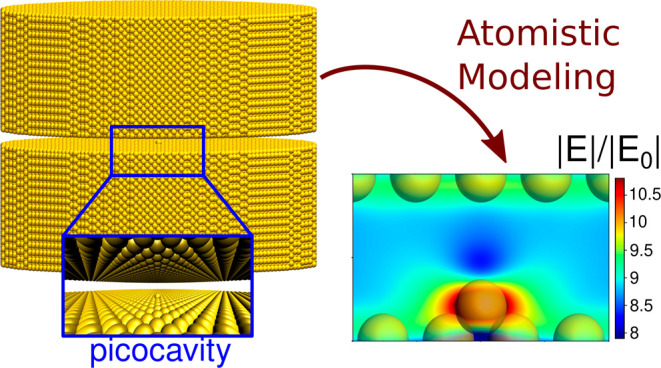

Picocavities are optical cavities confined to subnanometer volumes that have recently obtained significant attention due to their excellent ability to localize light into atomic-scale regions.^1–7^ Picocavities are generally constructed as single-atom defects—known as adatoms—on metallic surfaces typically composed of noble metals.^2^ Such structures are experimentally realized using the nanoparticle-on-mirror (NPoM) morphology,^8–11^ where a metallic nanoparticle (NP) is placed on top of a metal film separated by a molecular spacer layer, which is used to maintain a fixed NP-film distance. This way, a well-defined nanogap is created. However, it is worth noting that picocavities might also originate in plasmonic systems with less controlled morphology, including granular silver nanoparticle films and electrochemically roughened silver surfaces.^12^ When irradiated with an external electric field laser, picocavities allow for the confinement of visible light to picometer scales, with effective mode volumes below 1 nm^3^.^1,2,7^ Similar plasmonic architectures also enable the study of light–matter interactions on atomic scales, making picocavities an optical platform for applications in surface-enhanced Raman scattering (SERS),^1,7,13^ molecular electronics, and photocatalysis.^14^ This underscores the need for a physically grounded description of picocavity field morphology, which is essential for advancing our understanding of the coupling between light, plasmons, and matter at atomic dimensions. Indeed, as highlighted by Baumberger in ref 2, the recent advancements in the engineering of nanostructures at the atomic scale,^15–18^ as in the case of picocavities, demand novel theoretical approaches.

In this work, we exploit a theoretical model that addresses the current limits of the state-of-the-art methods^4,12,19,20^ in the prediction of the electric field morphology arising in picocavities upon plasmon excitation. The plasmonic response is modeled by exploiting the recently developed fully atomistic, yet classical approach named frequency-dependent fluctuating charges and fluctuating dipoles (ωFQFμ), which is specifically designed to describe the plasmonic response of metal nanostructures.^21,22^ ωFQFμ offers a remarkable accuracy that is comparable to state-of-the-art *ab initio* time-dependent density functional theory (TDDFT) even for structures below the quantum size limit^22^ and is also able to account for quantum effects thanks to an effective description of quantum tunneling.^21,23^ The high-level results provided by ωFQFμ question whether a quantum mechanical treatment is needed to describe the plasmonic response of metal nanostructures.^22^ Being based on a classical treatment of the nanostructure, ωFQFμ can be applied to nanostructure sizes that are untreatable at the *ab initio* level.^24^ Here, we consider an Au nanocavity (see Figure 1, top) composed of two metal disk plates (width: 19 nm, height: 6.5 nm, lattice constant: 4.08 Å^25^) with a distance of 1.0 nm, consistently with experimental setups.^2^ A plasmonic picocavity is constructed by inserting an atomic defect (adatom) in the first outer shell of one of the two metal plates (distance 2.04 Å from the plate, see Figure 1, bottom, and inset). This geometry directly resembles the NPoM experimentally exploited.^2^ Additionally, we model the same system with an adjacent vacancy near the adatom, mimicking a possible nonequilibrium scenario that may arise during picocavity formation (see Section S4.1 in the Supporting Information - SI). Remarkably, the studied Au nano/picocavitiy is composed of about 174000 atoms, a dimension that is far from the current limit of state-of-the-art methodologies.^26,27^ Our study aims to shed light on the morphology of the induced electric field in picocavities, which requires a fully atomistic modeling while treating large structures to avoid boundary effects affecting the optical response (see Figure 1).

**Figure 1:**
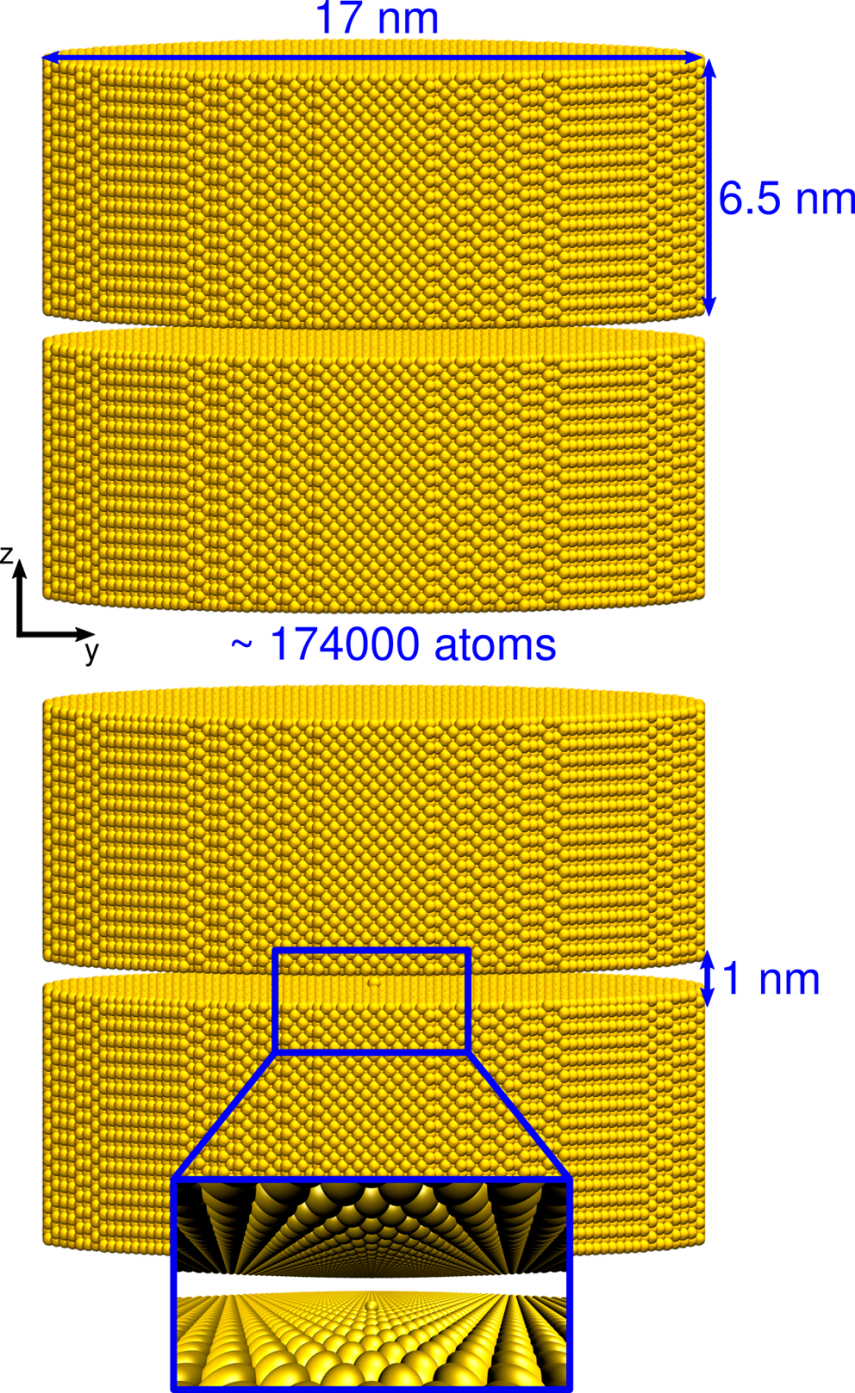
Graphical depiction of the nanocavity (top) and picocavity (bottom) considered in this work.

In ωFQFμ, each atom is endowed with a charge *q* and a dipole **μ** that respond to the external oscillating electric field.^21,28^ Charges are introduced to model intraband excitations;^21^ charge exchange between atoms is governed by the Drude mechanism, modulated by quantum tunneling effects, guaranteeing the correct plasmonic behavior at the nanogaps such as those created in picocavities.^22,23^ Dipoles are instead introduced to describe interband transitions, because *d*-electrons can be treated as localized polarizable shells placed at the atomic positions.^29^ Thanks to the physical coupling between the two effects, ωFQFμ can correctly model the plasmonic properties derived from the presence of *d*-states in noble metals, such as gold.^22^ Charges and dipoles represent the induced charge density, from which response properties (e.g., absorption cross section and induced field) can be calculated. More details on ωFQFμ are given in Section S1 in the SI.

ωFQFμ absorption cross sections for the nano/picocavity (Figure 1) obtained by applying an external electric field along the *z* direction are graphically depicted in Figure 2a (top: nanocavity; bottom: picocavity). As can be expected, the two spectra are perfectly superimposable (see also Figure S4a given as SI), since a single-atom defect does not affect the overall induced plasmonic response of such a large nanostructure. Both absorption spectra are characterized by a main peak at about 2.16 eV (∼574 nm, plasmon resonance frequency—PRF). To analyze the plasmonic response in the nanostructure, we compute the induced electric field enhancement |**E**|/|**E**_0_| in the middle of the gap between the two plates as a function of the incident frequency (see Figure 2a, blue line), which presents a maximum close to the plasmonic peak (2.12 eV). For both cavities, the maximum field enhancement is small (<10) because the studied structures are characterized by a relatively small size in the *z* direction (6.5 nm) and by the absence of sharp features in the region of interest, besides the adatom. However, this does not affect the electric field morphology within the gap. Notably, the field in the middle of the cavity differs in the two nanostructures. In particular, the picocavity-induced field in this region is slightly smaller than that generated by the nanocavity (see Figure S4b in the SI). This effect is surprising and has never been previously observed by other theoretical methods,^2,4,30^ which predict the electric field of a picocavity to be larger everywhere in the space close to the adatom. To deepen this point, we compute the induced density at the plasmon resonance frequency (see Figure 2b). For both structures (top, nanocavity; bottom, picocavity), the plasmonic peak is associated with the boundary dipolar plasmon (BDP) mode (using the nomenclature generally exploited in subnanometer junctions^31–36^ ), i.e. the two plates are characterized by an induced plasmon with dipolar character in the same direction (along the polarization field). Figure 2c provides a graphical depiction of the BDP induced density focusing on the atoms close to the gap in the middle *yz* plane containing the adatom (highlighted region in Figure 2c with green box). The nanocavity induced density (top panel) provides an enlarged picture of the aforementioned BDP distribution, with the two facing plates oppositely charged. Notably, the charge distribution is homogeneous among all the atoms. This physical result is obtained in our modeling because the two plates are large enough to avoid potential boundary effects. The presence of adatoms (Figure 2 c, bottom) induces drastic changes in the local induced density distribution. In fact, while the two facing plates overall conserve an induced density similar to the nanocavity (Figure 2c, top panel), the induced density close to the adatom presents a clear local dipolar character. Such a dipole is pronounced and also asymmetric (the charge density on top of the adatom is slightly larger than the opposite charge density). This is one of the main findings of the present work: a single atom defect induces a local, highly inhomogeneous induced density. This complex distribution of the induced density is the main reason for the different outcomes predicted by our approach and the previous results.^2,4,5,30^

**Figure 2:**
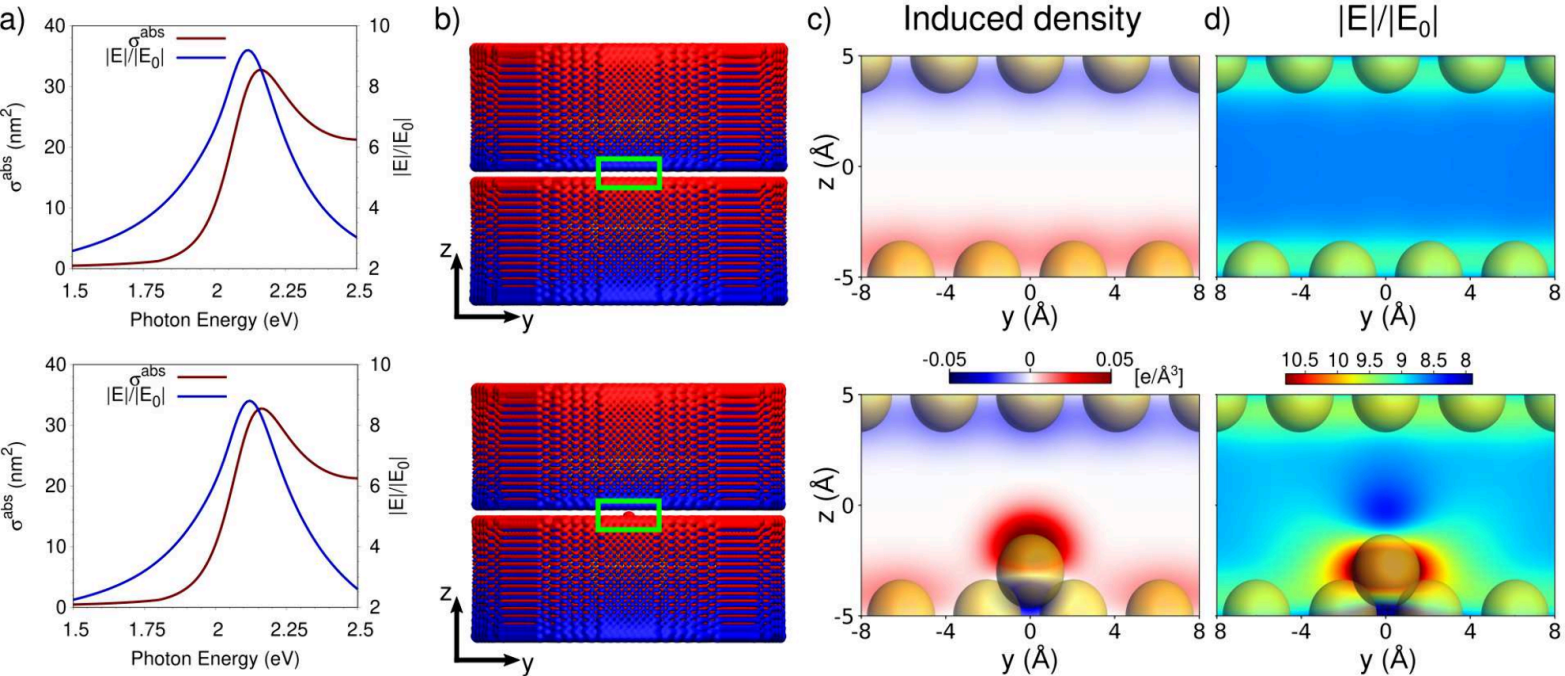
(a) ωFQFμ absorption cross sections σ^abs^ (nm^2^) and electric field enhancements (|**E**|/|**E**_0_|) calculated for the nanocavity (top) and picocavity (bottom). (b) Graphical depiction of nanocavity (top) and picocavity (bottom) induced density calculated at the plasmon resonance frequency (PRF). (c, d) 2D color map of nanocavity (top) and picocavity (bottom) induced density (c) and electric field enhancement (d) in a zoomed region in the middle *yz* plane (see green highlighted region in b panel). Gold atoms are shown using their van der Waals radius (1.66 Å).

The marked local different induced density in nano and picocavities is expected to affect all the related electronic properties drastically. In this work, we focus on the electric field morphology of picocavities. To analyze it, we graphically depict the electric field enhancement |**E**|/|**E**_0_| in the middle of the gap of both pico and nanocavities as color maps (see Figure 2d). This quantity is fundamental in molecular plasmonics because it is directly related to the coupling strength between the molecular and the plasmonic excitations.^37^ In turn, this enters several diverse phenomena, such as SERS signal enhancements of molecules adsorbed to the nanostructure, specifically its fourth power (|**E** ^4^|/|**E**_0_^4^|). Therefore, determining the electric field morphology permits us to highlight undisclosed ways in which single atomic defects can potentially affect light–matter interactions at the nanoscale. This emerges from the comparison between the induced field enhancements in nano- and picocavities. By focusing on Figure 2d top panel, we note that the homogeneous induced density in the nanocavity induces an enhancement of the electric field in the gap of about 8.5–9, which is almost constant in the nanogap. As expected from the plasmon distribution, this closely resembles the uniform electric field of a parallel plate capacitor, with minimal boundary effects due to the large nanostructure considered in this study. The electric field distribution induced by the picocavity closely resembles that of the nanocavity in the regions that are far apart from the adatom. However, in the immediate adatom proximity (see Figure 2d bottom panel), the induced field distribution becomes inhomogeneous, exhibiting a significant local variation. The field distribution is far from intuitive: it exhibits a cylindrical symmetry with a pronounced field enhancement near the van der Waals radius of the adatom (gold atoms in Figure 2d are shown using their van der Waals radius). Additionally, the presence of the adatom induces a marked field depletion immediately above it, in the region between the two cavity planes (as anticipated by the numerical |**E**|/|**E**_0_| values reported in Figure 2a). This constitutes one of the most important findings of our study, because it uncovers aspects that have not been identified by previous approaches.^2^

To better appreciate the local distortion induced by the adatom, Figure 3 shows the ratio between the field induced in the picocavity and that in the nanocavity. This plot enables a more detailed discussion of the differences observed in the two cases. In general, the picocavity exhibits a higher induced field at every point within the nanocavity (where the background color is dark pink), despite the enhancement being more pronounced and diminishing with distance from the adatom. The induced electric fields generated by the two nanostructures become nearly indistinguishable at a distance of approximately 24 Å from the adatom (see Figure S5 in the SI). Near the adatom, the cylindrical symmetry of the enhancement is intensified compared to that shown in Figure 2d, bottom panel). Furthermore, the anticipated depletion zone is clearly visible at the van der Waals radius of the adatom within the gap between the two planes (A zone in Figure 3). Remarkably, this region remains accessible to a physisorbed molecule, potentially leading to substantial modifications of its electronic structure. It should also be noted that Figure 4 makes evident the emergence of a second, more intense depletion zone within the plane that hosts the adatom (B zone in Figure 3), corresponding to the opposite density induced by the dipole on the atom in the induced density (see Figure 2c bottom panel). It is worth noting that the main features of the discussed findings are also reproduced in the nonequilibrium structure characterized by the vacancy (see Section S4.1 in the SI), confirming the general validity of our results.

**Figure 3:**
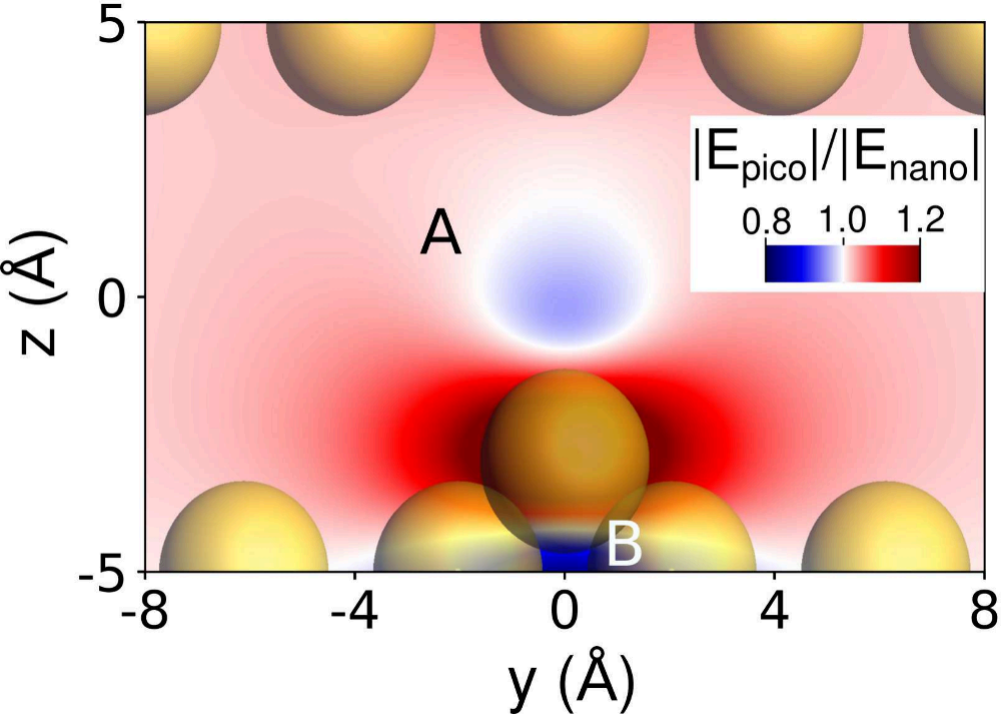
2D color map of the ratio between picocavity and nanocavity electric field enhancement in a zoomed region of the *yz* plane containing the picocavity adatom. A and B highlight the two depletion zones. Gold atoms are shown using their van der Waals radius (1.66 Å).

**Figure 4:**
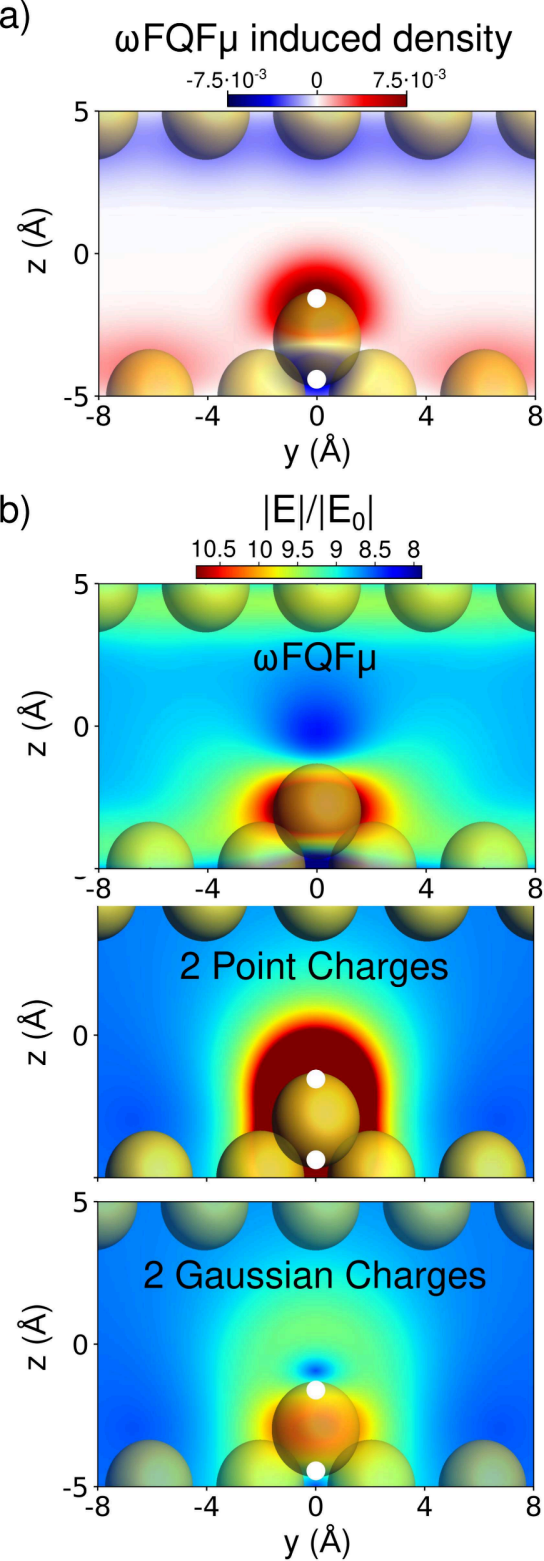
(a) 2D color map of picocavity induced density. (b) 2D color map of picocavity electric field enhancement computed at ωFQFμ level (top) and by using an analytical model representing the induced density as a plate capacitor and two point charges (middle) or two Gaussian-shaped charges (bottom), placed at the charge centroids (white points in panels a and b).

To understand the origins of this peculiar induced field behavior within the picocavity, we sought to identify the minimal physical characteristics responsible for a field morphology similar to that calculated at the ωFQFμ level. This approach allows us to uncover the underlying physical causes of this unique field distribution. By considering the distribution of the local induced density around the adatom, shown in Figure 4a, we first hypothesize that the system can be approximated by a dipole embedded within a constant field between two parallel capacitor plates.

To accurately represent the dipole calculated at the ωFQFμ level, we place two charges at the centroids of the charge density (shown as white points in Figure 4b, middle and bottom panels). As a first approximation, these two charges are modeled as point charges in the selected positions. The induced field from this minimal analytical model is displayed in Figure 4b, middle panel (see also Section S3 in the SI). As it is evident, the field obtained from this simplified approach significantly differs from the ωFQFμ-calculated field (also shown in Figure 4b, top panel for comparison). Specifically, the analytically calculated field shows a region of substantial enhancement near the adatom but lacks any depletion zones. Notably, this field closely resembles those derived from nonatomistic, implicit models reported in ref 2.

Unlike most classical models,^2,4,38–41^ charges and dipoles used to represent the ωFQFμ plasmonic response are characterized by a Gaussian, rather than point-like, distribution, consistent with their intrinsic quantum nature (see eqs S1 and S2 in the SI). We thus constructed a second minimal analytical model where the charges representing the quantum dipole of the adatom are endowed with a Gaussian distribution. The resulting field is shown in Figure 4. Comparison with the ωFQFμ reference reveals that the primary characteristics of the induced field are preserved: the field enhancement displays cylindrical symmetry around the atom along the *y*-axis. Additionally, a depletion zone emerges within the gap between the two plates—an effect that only appears when assuming a quantum-like (i.e., nonpoint-like) distribution of the electronic response densities of the structure. It is important to note, however, that the electric field enhancement calculated by using the second analytical model also presents some discrepancies from the ωFQFμ-calculated field, such as the elevated field intensity in regions surrounding the depletion zone. This indicates that while such a simple model can replicate some of the main features of a picocavity field, the atomic-level detail captured by ωFQFμ is significantly more complex and difficult to approximate through simplified models. This reinforces the necessity of using a fully atomistic approach like ωFQFμ for accurately characterizing picocavity fields.

The field distortions induced by the picocavity, particularly the highly localized enhancements and depletion zone A, can significantly influence the molecular electronic structure of nearby species, as evidenced by the experimental measurements.^2,12^ The strong localization of the optical plasmonic field near the adatom can create an effective potential landscape, dynamically confining the electronic density of a molecule and altering its spatial distribution. This optical confinement can result in shifts in the molecular energy levels, modifying the electronic excitation spectrum and potentially enhancing charge transfer interactions.^12^ Additionally, the depletion zone A within the plane hosting the adatom can lead to inhomogeneities in the local field environment as compared to the nanocavity. These can alter the coupling of specific molecular vibrational normal modes to the plasmonic field, modifying the corresponding spectral frequencies and intensities, especially in SERS.^6,12^ Experimental efforts in this direction can validate our theoretical predictions, as SERS is particularly sensitive to the electric field morphology, by comparing with theoretical SERS spectra computed at the hybrid Quantum Mechanical/ωFQFμ level.^42^ Finally, the peculiar electric field morphology can nontrivially influence molecular reactivity, as the selective coupling of specific vibrational modes to the picocavity-induced field distortions, in particular near their resonance frequencies, might lead to mode-specific heating or activation, thereby lowering reaction barriers or enhancing specific pathways.^43,44^

To conclude, we have presented the first detailed visualization and analysis of picocavities plasmonic induced fields, unraveling the atomistic features and patterns that emerge at the nanoscale and providing novel physical insights into the fundamental interactions governing nanophotonic behavior in these structures. Our results enhance our understanding of light confinement at the nanoscale and pave the way for future advancements in the in-silico design and application of picocavities. By offering a refined description of picocavity behavior, this model bridges the gap between experimental insights and theoretical predictions, advancing our ability to explore and harness atomic-scale light confinement.

Furthermore, this study elevates ωFQFμ as a powerful tool for nanophotonic research, thanks to its atomistic nature and the *ab initio* level accuracy while enabling the treatment of systems that are otherwise computationally unaffordable. Our method thus bridges the gap between computational efficiency and high accuracy, establishing as a unique tool for investigating the plasmonic response of complex nanostructures characterized by atomistic defects, such as picocavities. Through ωFQFμ, we can therefore anticipate uncovering novel phenomena and characteristics of picocavity-induced electric fields that were previously inaccessible due to the limits of the current theoretical methods. By capturing the morphology of induced electric fields in unprecedented detail, we contribute to the foundational knowledge necessary for optimizing nanophotonic devices and applications. Our approach allows for precise field enhancement in atomistically defined geometries, offering a pathway to the design of more efficient substrates for molecular sensing^1,37,45–50^ and related applications, such as plasmonic catalysis.^43,51–60^

## Supplementary Material



## Notes

ωFQFμ model; computational details; analytical model; ωFQFμ results on picocavity and adjacent vacancy (PDF)
