# Correction to “The Electric Field Morphology of Plasmonic Picocavities”

**DOI:** 10.1021/acs.nanolett.6c04064

**Published:** 2026-09-28

**Authors:** Tommaso Giovannini, Luca Nicoli, Stefano Corni, Chiara Cappelli

In the original publication, in eqs S7 and S8 in the Supporting Information, the sign of the dipole contribution to the ωFQFμ induced electric potential and field is incorrect. The correct formula for the induced electric potential (eq S7 in the SI) at a generic point **r**_*j*_ reads as follows:

1 $$V_{X}\left(r_{j}\right)=\overset{N_{atoms}}{\underset{i}{\sum }}q_{i}^{X}\left[\frac{1}{\left|r_{ij}\right|}\mathrm{erf}\left(\frac{\left|r_{ij}\right|}{R_{q_{i}}}\right)\right]-\mu_{i}^{X}\left[\frac{r_{ij}}{|r_{ij}{|}^{3}}\left(\mathrm{erf}\left(\frac{\left|r_{ij}\right|}{R_{\mu_{i}}}\right)-\frac{2\left|r_{ij}\right|}{\sqrt{\pi }R_{\mu_{i}}}\exp\left(-\frac{|r_{ij}{|}^{2}}{R_{\mu_{i}}^{2}}\right)\right)\right]$$

The induced electric field at a generic point **r**_*j*_ can be calculated as

2 EX(rj)=−∑iNatomsqiX[rij|rij|3(erf(|rij|Rqi)−2|rij|πRqiexp(−|rij|2Rqi2))]+μiX(3rij⊗rij−|rij|2I|rij|5[erf(|rij|Rμi)−2π|rij|Rμiexp(−|rij|2Rμi2)]−4πRμi3rij⊗rij|rij|2exp(−|rij|2Rμi2))

Note that the plasmonic responses of the nanostructure, i.e,. the induced charges *q*_*i*_, the induced dipoles μ_*i*_, the induced density, and the absorption cross sections, remain unchanged because the original error in sign only affects the evaluation of the field from the (correctly computed) charges and dipoles. The error in sign in the original paper affects the numerical values of the induced electric field maps reported in Figures 2a and d, 3, and 4 of the original paper, whose corrected versions are below. The corrected versions of the corresponding figures in the Supporting Information (SI) (Figures [Fig figS2], [Fig figS3], [Fig figS4], and [Fig figS5]) are below. The corrected graphical TOC is also here.
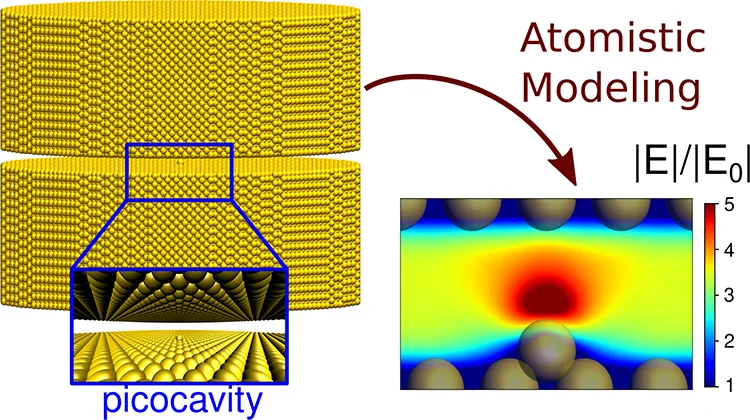


**Figure 2:**
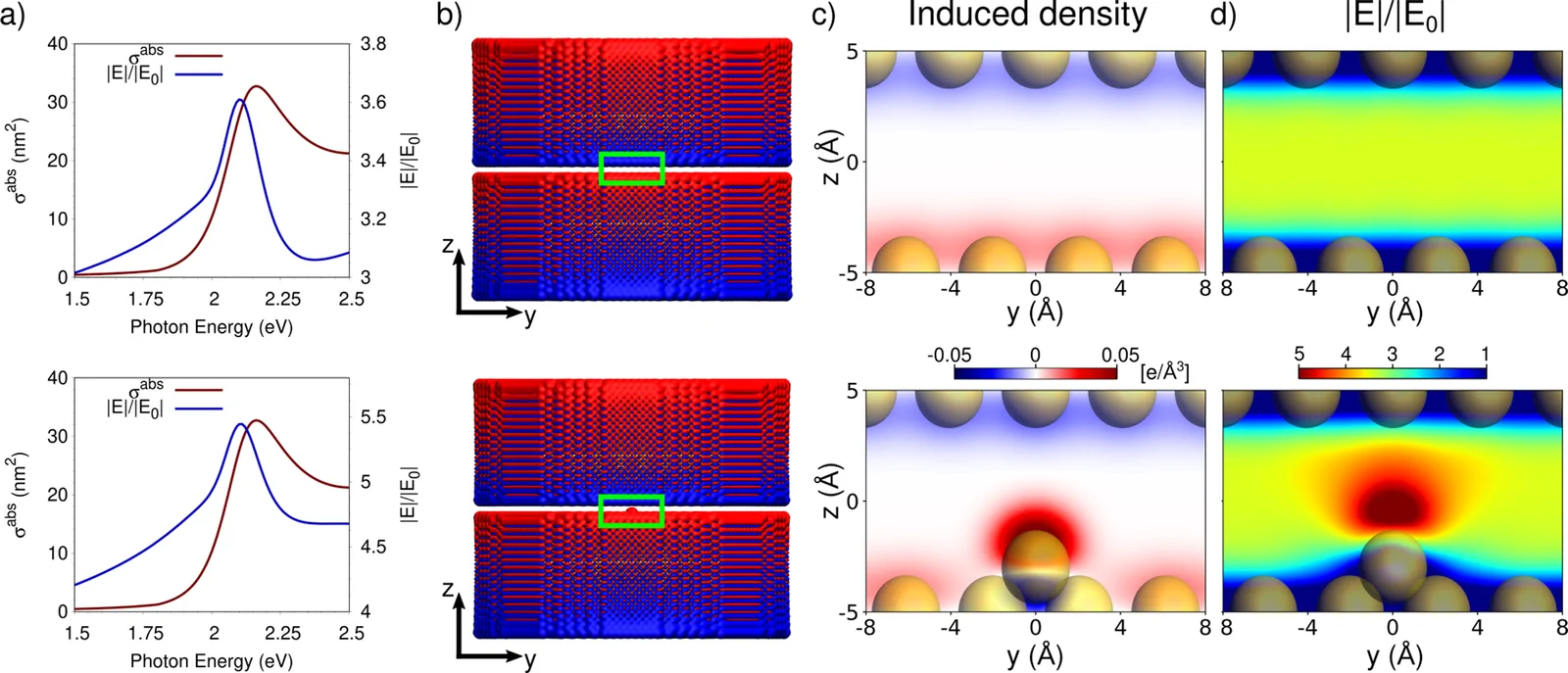
(a) ωFQFμ absorption cross sections σ^abs^ (nm^2^) and electric field enhancements (|**E**|/|**E**_0_|) calculated for the nanocavity (top) and picocavity (bottom), (b) Graphical depiction of nanocavity (top) and picocavity (bottom) induced density calculated at the plasmon resonance frequency (PRF), (c, d) 2D color map of nanocavity (top) and picocavity (bottom) induced density (c) and electric field enhancement (d) in a zoomed region in the middle *yz* plane (see green highlighted region in b panel). Gold atoms are shown using their van der Waals radius (1.66 Å). Panels (b) and (c) are unchanged. Panels (a) and (d) are computed with the corrected sign of the dipole contribution to the induced field.

**Figure 3:**
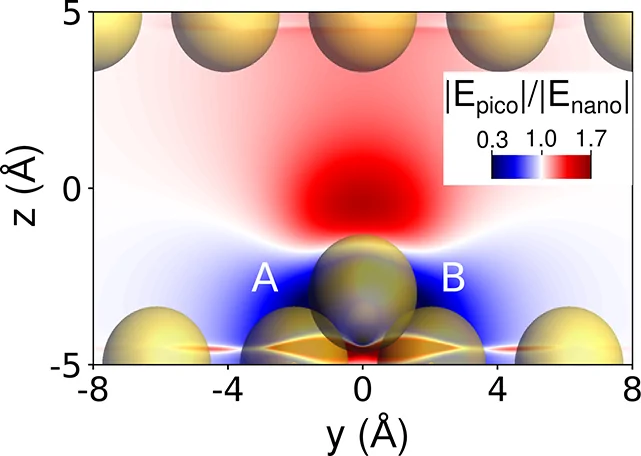
2D color map of the ratio between picocavity and nanocavity electric field enhancement in a zoomed region of the *yz* plane containing the picocavity adatom. A and B highlight the two depletion zones. Gold atoms are shown using their van der Waals radius (1.66 Å).

**Figure 4:**
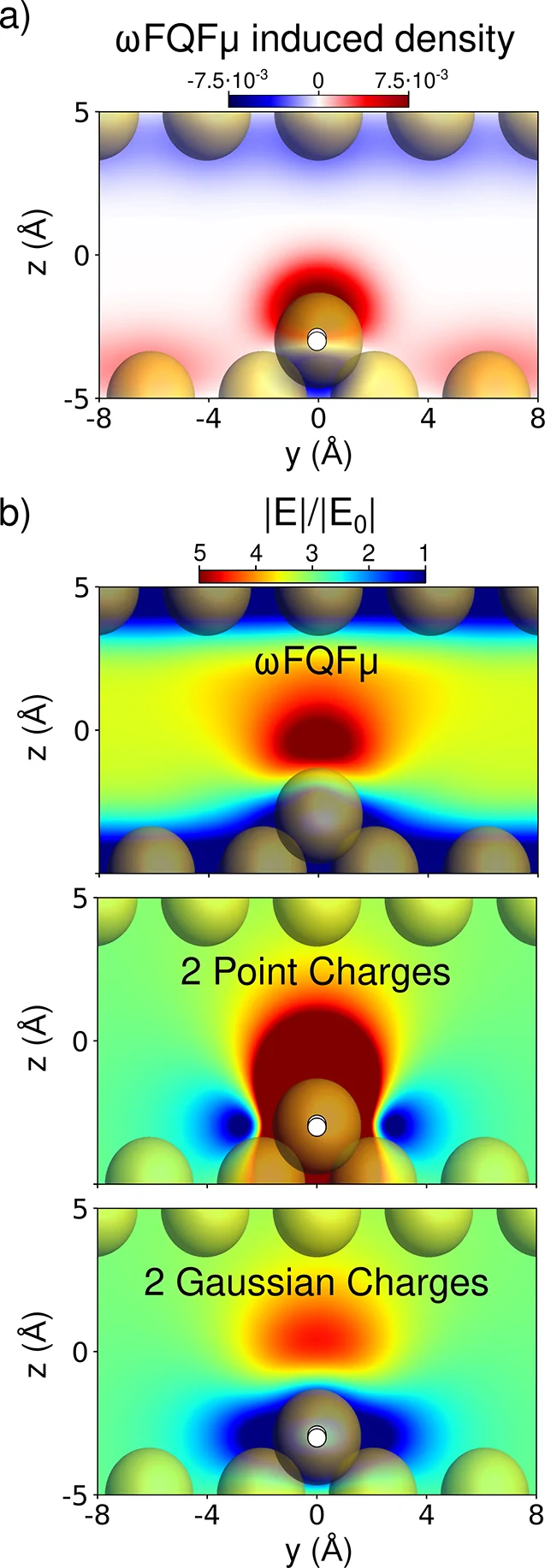
(a) 2D color map of picocavity induced density. (b) 2D color map of picocavity electric field enhancement computed at ωFQFμ level (top) and by using an analytical model representing the induced density as a plate capacitor and two point charges (middle) or two Gaussian-shaped charges (bottom), placed at the white points in panels (a) and (b). Panel (a) is unchanged. Panel (b) is computed with the corrected sign of the dipole contribution to the induced field. Panels (c) and (d) are computed using the parameters reported in the corrected Table S1.

**S2 figS2:**
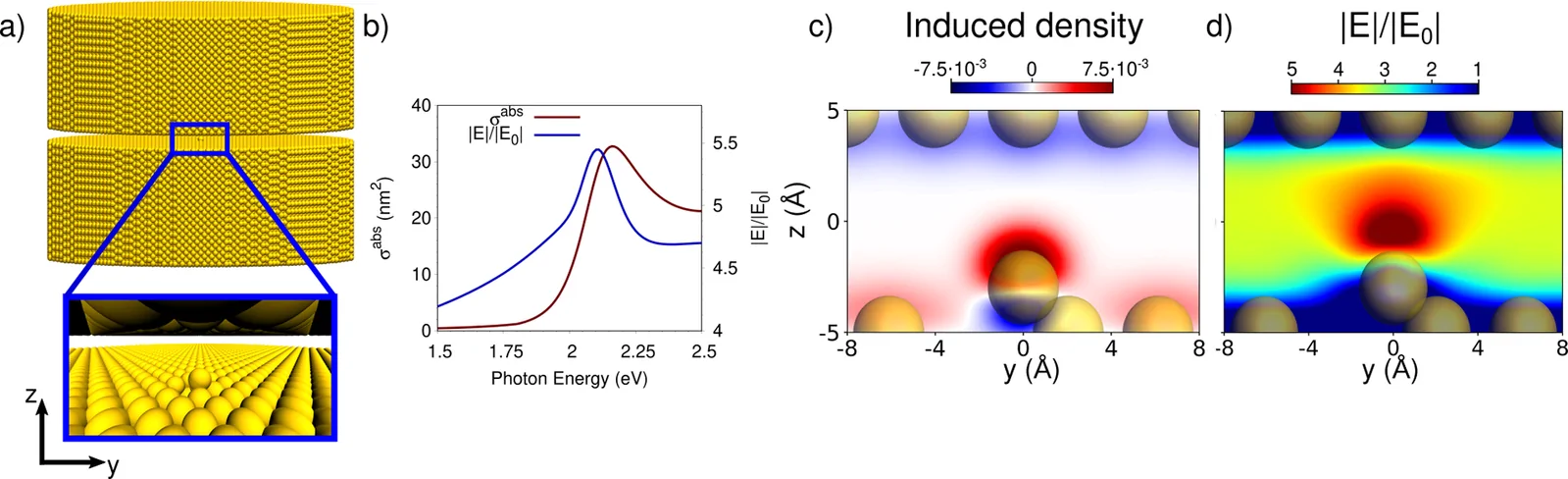
(a) Graphical depiction of the picocavity with vacancy considered in this work. (b) ωFQFμ absorption cross sections σ^abs^ (nm^2^) and electric field enhancements (|**E**|/|**E**_0_|) calculated for the considered nanocavity (top) and picocavity (bottom). (c, d) 2D color map of picocavity with vacancy (bottom) induced density (c) and electric field enhancement (d) in a zoomed region in the middle *yz* plane of the nanostructure (see green highlighted region in b panel). Gold atoms are shown using their van der Waals radius (1.66 Å). Panels (b) and (c) are unchanged. Panes (a) and (d) are computed with the corrected sign of the dipole contribution to the induced field.

**S3 figS3:**
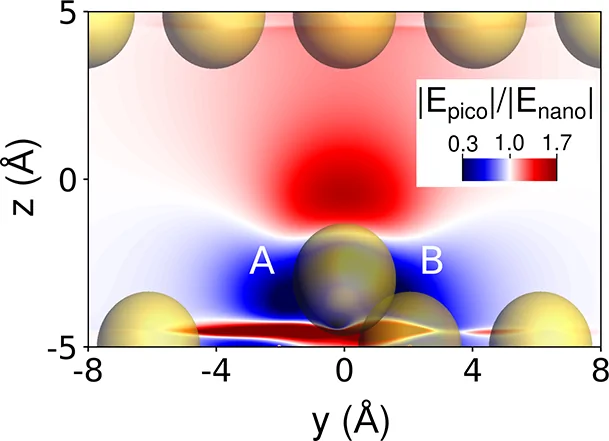
2D color map of the ratio between picocavity with vacancy and nanocavity electric field enhancement in a zoomed region of the *yz* plane containing the picocavity adatom. A and B highlight the two depletion zones. Gold atoms are shown using their van der Waals radius (1.66 Å).

**S4 figS4:**
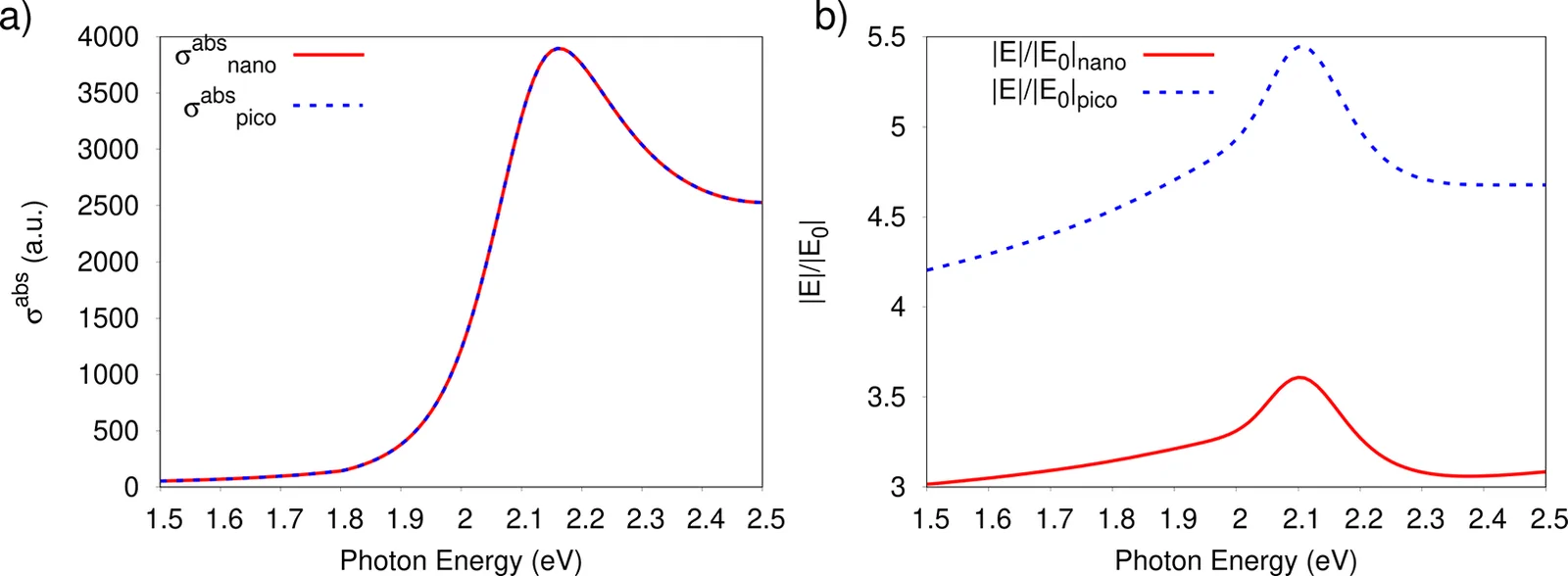
ωFQFμ (a) absorption cross sections σ^abs^ and (b) electric field enhancements (|**E**|/|**E**_0_|) calculated for the nanocavity (nano) and picocavity (pico). Panel (a) is unchanged. Panel (b) is computed with the corrected sign of the dipole contribution to the induced field.

**S5 figS5:**
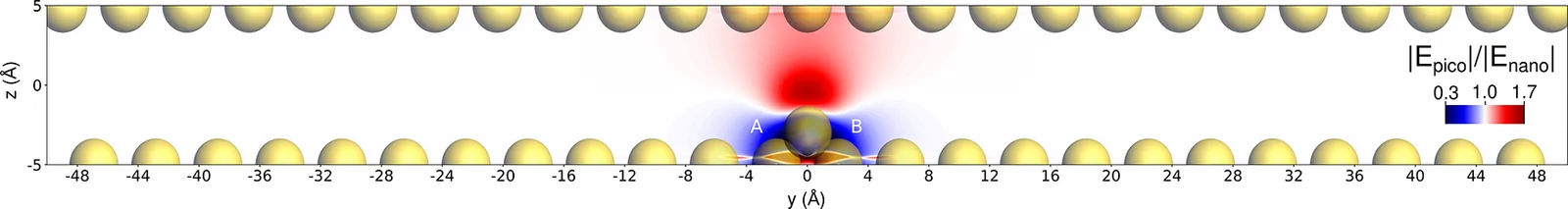
2D color map of the ratio between picocavity and nanocavity electric field enhancement in a large region of the *yz* plane containing the picocavity adatom. A and B highlight the two depletion zones. Gold atoms are shown using their van der Waals radius (1.66 Å).

The relevant corrections to the original paper and the SI are summarized in Table 1.

**Table 1:** Summary of Corrections to the Original Paper and SI (all other results, figures, and conclusions are unaffected)

Position	Correction
Figure 2(d) and caption	Replace with the corrected induced-field map (Figure 2).
Figure 3 and caption	Replace with the corrected ratio map (Figure 3).
Figure 4(b) and caption	Replace with the corrected ωFQFμ/analytical field maps (Figure 4).
Discussion of zones A and B	After the sign correction, the field morphology along and perpendicular to the dipole axis is inverted: the on-axis regions immediately above and below the adatom, originally described as depletion zones, now exhibit a field enhancement, while the two depletion zones (A and B) develop in the equatorial region, lateral to the dipole axis. The two-depletion-zone picture is retained but its spatial arrangement is inverted.
Eqs S7 and S8 (SI)	The dipole contribution carries the opposite sign, as in eqs 1 and 2.
Figures [Fig figS2], [Fig figS3], [Fig figS4], [Fig figS5] (SI)	Replace with the corrected maps (Figures S2–S5).
Graphical TOC	Replace with the corrected maps (TOC graphic).

We remark that the central physical conclusions of the original work remain valid: a single-atom defect produces a strongly inhomogeneous induced field, and the picocavity field morphology cannot be reproduced by nonatomistic models. In particular, the two depletion zones (A and B) are still predicted, so the qualitative picture and the main message of the paper are unchanged. The main differences are associated with the morphology of the depletion. Upon correcting the sign of the dipole contribution, the field morphology along and perpendicular to the dipole axis is essentially inverted. In the original figures, the region along the dipole axis immediately above and below the adatom appeared as a field depletion (zones A and B), while the equatorial region showed a field enhancement. After the correction, the same on-axis regions instead exhibit a field enhancement, and the two depletion zones (A and B, see Figure 3) develop in the equatorial region, lateral to the dipole axis. Importantly, this corrected morphology can also be obtained by using the simplified analytical model by approaching the charges and increasing their value toward their dipolar limit (the new parameters for the calculations are reported in the corrected Table [Table tblS1] below).

**S1 tblS1:** Parameters Used in the Analytical Model

Parameter	Symbol	Value
Positive charge position	**r**_+_	(*x*_+_, *y*_+_) = (0.0, −2.91 Å)
Negative charge position	**r**_–_	(*x*_–_, *y*_–_) = (0.0, −3.01 Å)
Positive charge magnitude	*q*_+_	+62.0 au
Negative charge magnitude	*q*_–_	–62.0 au
Gaussian width	*W*_*q*_	2.1 Å
Background electric field strength	*E*_0_	3.0 au

The sentences in the original text describing the depletion zones A and B as located “immediately above” and “below” the adatom along the field direction should be read, after the correction, as referring to the equatorial field reduction described above (see also Table 1).

Finally, it is worth highlighting that the field morphology around the adatom is frequency dependent. While the corrected results discussed above refer to the plasmon resonance frequency, at other frequencies the depletion zone above the adatom, originally predicted at resonance, can indeed be recovered. As an example, in our new Figure [Fig figX], we report the ωFQFμ induced density and field enhancement computed for the nanocavity (top) and picocavity (bottom) at ω = 6.48 eV. At this frequency, a clear depletion region above the adatom is predicted (zone A in Figure [Fig figX]c), consistent with the original discussion. This confirms that the qualitative picture originally reported is physically meaningful and can indeed be observed. However, its occurrence depends on the excitation frequency rather than being a specific feature arising at the plasmon resonance.

**X figX:**
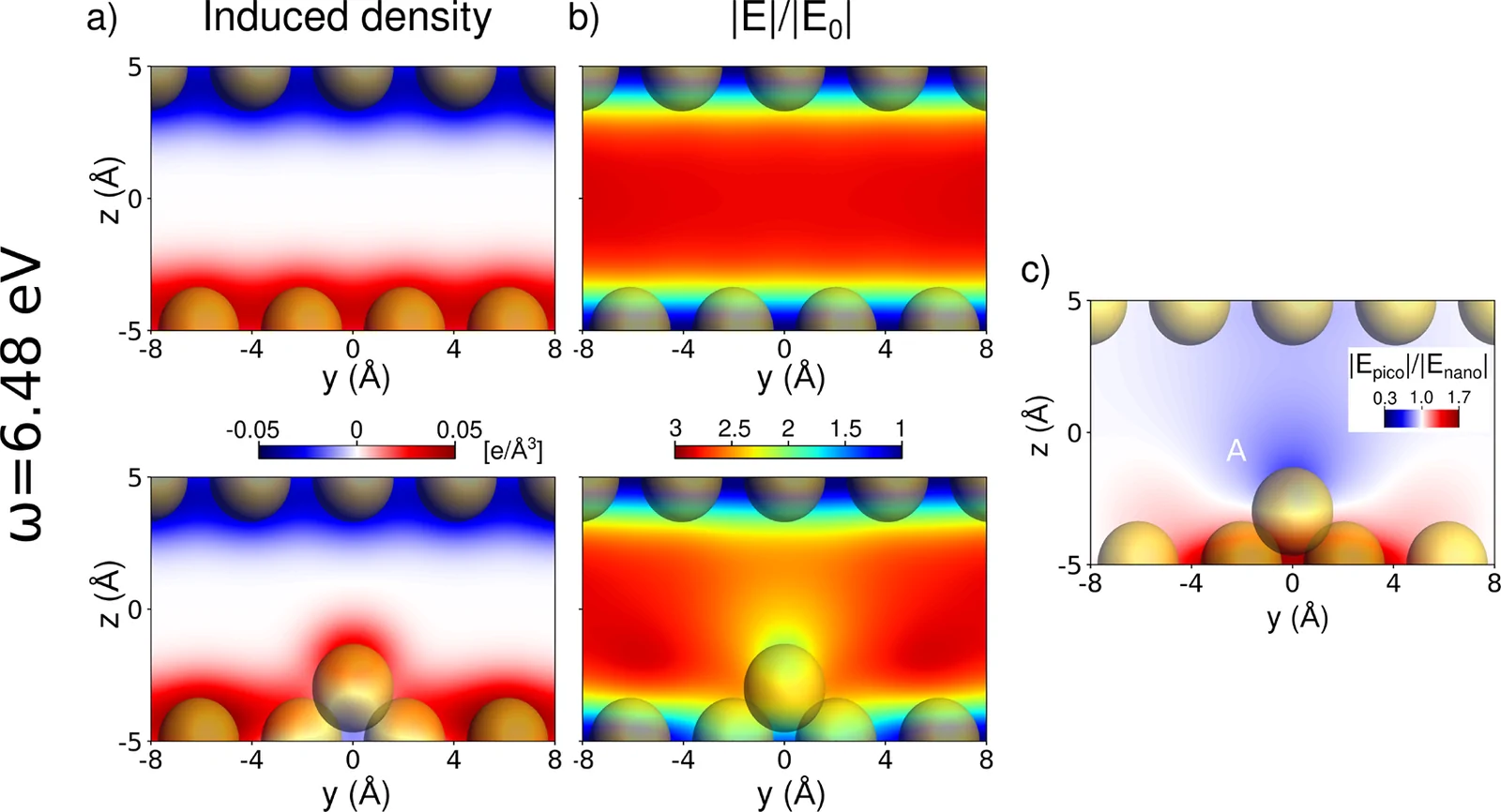
ωFQFμ (a) induced density and (b) induced electric field enhancement |**E**|/|**E**_0_| for the nanocavity (top) and picocavity (bottom), computed at ω = 6.48 eV; (c) ratio between the picocavity and nanocavity field enhancement, |**E**_pico_|/|**E**_nano_|, in a zoomed region of the *yz* plane containing the adatom. A denotes the depletion zone above the adatom.

