# Supplementary material for: The Electric Field Morphology of Plasmonic Picocavities
Source: Nano Lett. 2025 Jun 25;25(27):10802–8. doi: 10.1021/acs.nanolett.5c01999 (PMC12257643; doi:10.1021/acs.nanolett.5c01999)
Supplement: Supplementary file 1 [file nl5c01999_si_001.pdf]

Supporting Information for: The Electric Field Morphology of Plasmonic Picocavities

Tommaso Giovannini,[*,†] Luca Nicoli,[‡] Stefano Corni,[¶,§] and Chiara Cappelli[‡,‖]

†*Department of Physics and INFN, University of Rome Tor Vergata, Via della Ricerca Scientifica 1, 00133, Rome, Italy*

‡*Scuola Normale Superiore, Piazza dei Cavalieri 7, 56126 Pisa, Italy*

¶*Department of Chemical Sciences, University of Padova, via Marzolo 1, Padova, Italy*

§*CNR Institute of Nanoscience, via Campi 213/A, Modena, Italy*

‖*IMT School for Advanced Studies Lucca, Piazza San Francesco 19, Lucca 55100, Italy*

E-mail: tommaso.giovannini@uniroma2.it

S1 The ωFQFμ model

In ωFQFμ each atom is endowed with a charge q and a dipole $\boldsymbol{\mu}$ that respond to the external oscillating electric field.[1,2] To model a physically grounded induced density ρ, the charges and dipoles are associated with a Gaussian-type distribution as follows:[3,4]

$$\rho_{q_i}(\mathbf{r}) = \frac{q_i}{\pi^{3/2}R_{q_i}^3} \exp\left(-\frac{|\mathbf{r}-\mathbf{r}_i|^2}{R_{q_i}^2}\right) \tag{S1}$$

$$\rho_{\mu_i}(\mathbf{r}) = \frac{|\boldsymbol{\mu}_i|}{\pi^{3/2}R_{\boldsymbol{\mu}_i}^3} \hat{n}_i \cdot \nabla_{\mathbf{r}_i} \left[\exp\left(-\frac{|\mathbf{r}-\mathbf{r}_i|^2}{R_{\boldsymbol{\mu}_i}^2}\right)\right] \tag{S2}$$

where $\mathbf{r}_i$ is the i-th atom position, and R_{q_i} and R_{μ_i} are the i-th charge and dipole Gaussian widths, respectively. In the frequency domain, the ωFQFμ equation of motion for charges reads:[3]

$$\begin{aligned} -i\omega q_i(\omega) &= \frac{2n_0\tau}{1-i\omega\tau} \sum_j \left[1-f(l_{ij})\right] \frac{\mathscr{A}_{ij}}{l_{ij}} (\phi_j^{el} - \phi_i^{el}) \\ &= \sum_j K_{ij} (\phi_j^{el} - \phi_i^{el}) \end{aligned} \tag{S3}$$

where the complex-valued $q_i(\omega)$ is the Fourier component at the frequency ω of the oscillating atomic charge on atom i. τ is the scattering time, n_0 the electronic density, l_{ij} is the distance between i-th j-th atoms, and $\mathscr{A}_{ij}$ is the effective area connecting the two atoms. ϕ_i^{el} is the electrochemical potential acting on the i-th metal atom, which takes into account the interactions between the charges and dipoles placed on different atoms and the interaction with the potential associated with the external electric field. $f(l_{ij})$ is a Fermi-like function that mimics quantum tunneling effects:[1]

$$f(l_{ij}) = \frac{1}{1+\exp\left[-d\left(\frac{l_{ij}}{s\cdot l_{ij}^0}-1\right)\right]} \tag{S4}$$

where l_{ij}^0 is the nearest neighbor's distance, which for Au in face-centered cubic (FCC) is equal to 2.88 Å.[5,6] The parameters d and s determine the sharpness and the center of the step function, respectively, and are determined by comparison with first-principles results.[3]

The induced dipole moments $\boldsymbol{\mu}_i$ are obtained by solving the following set of linear equations:

$$\boldsymbol{\mu}_i = \alpha_i^{\omega} \left(\mathbf{E}_i^{ext} + \mathbf{E}_i^{\mu} + \mathbf{E}_i^{q} \right) \tag{S5}$$

where $\mathbf{E}^{ext}$, $\mathbf{E}^{\mu}$ and $\mathbf{E}^{q}$ are the external electric field, the field generated by the other dipole moments, and the field arising from all the other charges, respectively. The interband transitions can effectively be modeled due to the introduction of the atomic complex polarizability, α^{ω}, which is obtained by extracting the interband contribution to the experimental permittivity function.[3,7] Remarkably, ωFQFμ parameters can be defined in terms of physical quantities (experimental permittivity) or fitted against *ab initio* calculations (see Ref. 3 for the parametrization procedure). Due to its full atomistic nature and the proper inclusion of the decay mechanisms of plasmonic excitations, ωFQFμ substantially differs from classical approaches that solve Maxwell's equations for an approximated, non-atomistic description of the nanostructure, such as Discrete Dipole Approximation (DDA),[8] Boundary Element Method (BEM),[9–12] and Finite-difference time-domain (FDTD)[13] method. Furthermore, it is worth noting that the model can be applied to very large nanostructures composed of thousands/millions of atoms.[14]

In order to couple the intraband (Drude) and the interband mechanisms, Eqs. S3 and S5 are solved simultaneously, yielding a set of linear equations that reads:

$$\left[\begin{pmatrix} \mathbf{A}^{qq} & \mathbf{A}^{q\mu} \\ T^{\mu \mathrm{q}} & T^{\mu\mu} \end{pmatrix} - \begin{pmatrix} z(\omega)\mathbf{I}_N & 0 \\ 0 & z'(\omega)\mathbf{I}_{3N} \end{pmatrix} \right] \begin{pmatrix} \mathbf{q} \\ \boldsymbol{\mu} \end{pmatrix} = \begin{pmatrix} \mathbf{R} \\ -\mathbf{E}^{\mathrm{ext}} \end{pmatrix} \tag{S6}$$

where $\mathbf{A}^{qq}$, $\mathbf{A}^{q\mu}$, $\mathbf{T}^{\mu q}$, $\mathbf{T}^{\mu\mu}$ represent the charge-charge, charge-dipole, dipole-charge, and dipole-dipole interaction kernels, respectively. The frequency dependence is gathered in the z and z' terms, and $\mathbf{I}_N$ is the NxN identity matrix (N is the number of atoms). The external potential and external field effects are included in the right-hand-side matrices $\mathbf{R}$ and $\mathbf{E}^{\mathrm{ext}}$. Calculating the plasmonic response of a nanostructured material corresponds to solving Eq. S6, i.e. a complex-linear system of $4N$ variables. Advanced computational techniques can be used to this end,[14] allowing for the efficient calculation of plasmonic properties of systems of unprecedented dimensions, as the one considered in this study (see Fig. 1 in the main text).

S1.1 ωFQFμ induced electric field

The electric potential induced by an external field in the X direction calculated at a generic point $\mathbf{r_j}$ reads:

$$V_X(\mathbf{r}_j) = \sum_i^{N_{atoms}} q_i^X \left[\frac{1}{|\mathbf{r}_{ij}|} \mathrm{erf}\left(\frac{|\mathbf{r}_{ij}|}{R_{q_i}} \right) \right] + \mu_i^X \left[\frac{\mathbf{r}_{ij}}{|\mathbf{r}_{ij}|^3} \left(\mathrm{erf}\left(\frac{|\mathbf{r}_{ij}|}{R_{\mu_i}} \right) - \frac{2\,|\mathbf{r}_{ij}|}{\sqrt{\pi} R_{\mu_i}} \exp\left(-\frac{|\mathbf{r}_{ij}|^2}{R_{\mu_i}^2} \right) \right) \right] \tag{S7}$$

where $\mathbf{r}_{ij}$ is the vector connecting atom i and point j, whereas X indicates real (Re) and imaginary (Im) parts of the electric potential and polarizable sources (charges/dipoles). The induced electric field at a generic point $\mathbf{r}_j$ can be calculated as follows:

$$\begin{aligned}\mathbf{E}_X(\mathbf{r}_j) = -\sum_i^{N_{atoms}} q_i^X & \left[\frac{\mathbf{r}_{ij}}{|\mathbf{r}_{ij}|^3} \left(\mathrm{erf}\left(\frac{|\mathbf{r}_{ij}|}{R_{q_i}} \right) - \frac{2\,|\mathbf{r}_{ij}|}{\sqrt{\pi} R_{q_i}} \exp\left(-\frac{|\mathbf{r}_{ij}|^2}{R_{q_i}^2} \right) \right) \right] \\ & - \mu_i^X \left(\frac{3\mathbf{r}_{ij} \otimes \mathbf{r}_{ij} - |\mathbf{r}_{ij}|^2 \mathbf{I}}{|\mathbf{r}_{ij}|^5} \left[\mathrm{erf}\left(\frac{|\mathbf{r}_{ij}|}{R_{\mu_i}} \right) - \frac{2}{\sqrt{\pi}} \frac{|\mathbf{r}_{ij}|}{R_{\mu_i}} \exp\left(-\frac{|\mathbf{r}_{ij}|^2}{R_{\mu_i}^2} \right) \right] \right. \\ & \left. - \frac{4}{\sqrt{\pi} R_{\mu_i}^3} \frac{\mathbf{r}_{ij} \otimes \mathbf{r}_{ij}}{|\mathbf{r}_{ij}|^2} \exp\left(-\frac{|\mathbf{r}_{ij}|^2}{R_{\mu_i}^2} \right) \right) \end{aligned} \tag{S8}$$

The total induced electric field is then calculated as:

$$\mathbf{E}(\mathbf{r}_j) = \sqrt{\mathbf{E}_{Re}^2(\mathbf{r}_j) + \mathbf{E}_{Im}^2(\mathbf{r}_j)} \tag{S9}$$

S2 Computational details

ωFQFμ equations[3] have been implemented in a Fortran2008 code, named plasmonX, which will be released open-source in the near future. ωFQFμ equations are solved by exploiting the GMRES algorithm as in Ref. 14, by imposing a Root Mean Square Error (RMSE) equal to 10^{-5}. Plasmon densities and induced fields are calculated by summing charge and dipole contributions (see eqs. S1 and S2 for plasmon densities and eqs. S8 and S9 for the electric field) computed at the plasmon resonance frequency. All Au NP structures have been constructed using the ASE python package, by imposing the lattice constant to be 4.08 Å in a Face-Centered Cubic (FCC) packing.[6]

S3 Analytical Model

The analytical model is based on the superposition of two electric field contributions:

1. *A uniform field generated by a parallel plate capacitor.* A capacitor with parallel plates generates a homogeneous electric field $\mathbf{E}_c$ in the region between the plates, oriented along the y-direction:

$$\mathbf{E}_c = \begin{cases} E_0\hat{\mathbf{y}}, & -D/2 < y < D/2 \\ 0, & \text{otherwise} \end{cases}$$

where E_0 is the magnitude of the uniform field, and D is the plate separation (10Å).

2. *A dipolar field produced by two charges localized at the charge centroids* (as highlighted in white in Fig. 4 in the main text). The two charges are treated as point-like or endowed with a Gaussian charge smearing.

(a) The electric field at a given grid point $\mathbf{r} = (x, y)$ due to a dipole constituted of two point charges, q_+ at $\mathbf{r}_+ = (x_+, y_+)$ and q_- at $\mathbf{r}_- = (x_-, y_-)$, is given by the superposition principle:

$$\mathbf{E}_d(\mathbf{r}) = \left[\frac{q_+(x-x_+)\hat{\mathbf{x}} + q_+(y-y_+)\hat{\mathbf{y}}}{d_+^3} + \frac{q_-(x-x_-)\hat{\mathbf{x}} + q_-(y-y_-)\hat{\mathbf{y}}}{d_-^3}\right] \tag{S10}$$

where d_+ and d_- are the distances of the grid point from the positive and negative charges, respectively; $\hat{\mathbf{x}}$ and $\hat{\mathbf{y}}$ are the unit vectors along the x- and y-directions.

(b) To account for a Gaussian charge distribution mimicking the quantum electron distribution, we introduce the spatial smearing function (see also Eq. S8):

$$T(r_q, W_q) = \text{erf}\left(\frac{r_q}{W_q}\right) - \frac{2r_q}{\sqrt{\pi}W_q}e^{-r_q^2/W_q^2}$$

where r_q is the charge position and W_q is the charge gaussian width. In this case, the dipole field components at a grid point $\mathbf{r} = (x, y)$ read:

$$E_{d,x} = T(r_+, W_q)\frac{q_+(x-x_+)}{d_+^3} + T(r_-, W_q)\frac{q_-(x-x_-)}{d_-^3}$$

$$E_{d,y} = T(r_+, W_q)\frac{q_+(y-y_+)}{d_+^3} + T(r_-, W_q)\frac{q_-(y-y_-)}{d_-^3}$$

The total electric field is given by the superposition of the capacitor and dipole field, i.e.:

$$\mathbf{E}_{\text{tot}} = \mathbf{E}_c + \mathbf{E}_d$$

The parameters exploited in the analytical model used in Fig. 4 (main text) are summarized in Tab. S1:

Table S1: Parameters used in the analytical model

**Parameter**	**Symbol**	**Value**
Positive charge position	$\mathbf{r}_+$	(x_+, y_+) = (0.0, 3.735 Å)
Negative charge position	$\mathbf{r}_-$	(x_-, y_-) = (0.0, 2.185 Å)
Positive charge magnitude	q_+	$+20.0$ a.u.
Negative charge magnitude	q_-	-20.0 a.u.
Gaussian width	W_q	2.1 Å
Background electric field strength	E_0	8.5 a.u.

To evaluate the dependence of the analytical model (b) on the Gaussian width W_q, we compute the electric field enhancement by varying W_q from 1.5 Å to 2.4 Å in constant steps of 0.1 Å. The results are shown in Fig. S1. While the amplitude of the electric field enhancement depends on the numerical value of W_q, the overall spatial distribution of the field remains only slightly affected. Importantly, the depletion zone located above the adatom is predicted in all cases. As W_q increases, such a depletion region shifts progressively farther from the adatom. $W_q = 2.1$ Å is used in Fig. 4 (main text) as it qualitatively resembles ωFQFμ results, although the simplified analytical model cannot fully reproduce the reference field distribution.

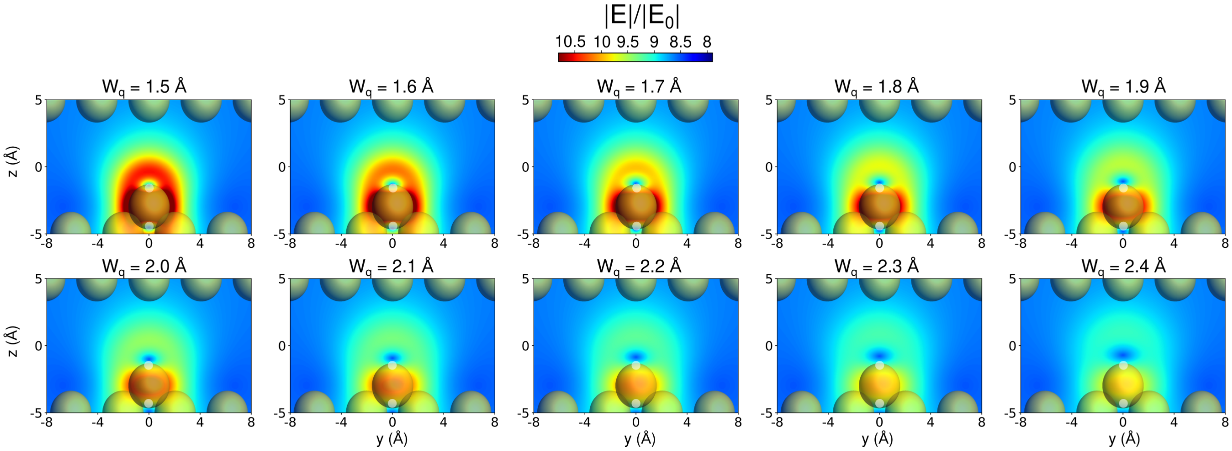

Figure S1: 2D color map of picocavity electric field enhancement computed by using the analytical model representing the induced density as a plate capacitor and 2 Gaussian-shaped charges (b model), placed at the charge centroids (white points in all panels). The electric field enhancements are computed for various Gaussian widths W_q (from 1.5 Å to 2.4 Å).

S4 Numerical Results

S4.1 Picocavities: Adatom and adjacent vacancy

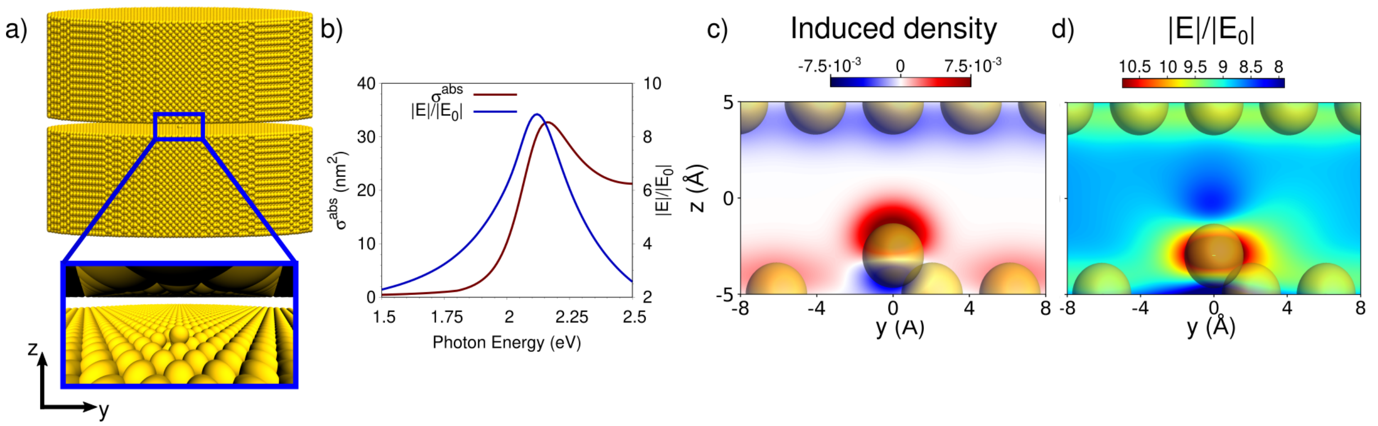

Figure S2: (a) Graphical depiction of the picocavity with vacancy considered in this work. (b) ωFQFμ absorption cross sections σ^{abs} (nm^2) and electric field enhancements ($|\mathbf{E}|/|\mathbf{E}_0|$) calculated for the considered nanocavity (top) and picocavity (bottom); (c-d)2D color map of picocavity with vacancy (bottom) induced density (c) and electric field enhancement (d) in a zoomed region in the middle yz plane of the nanostructure (see green highlighted region in b panel). Gold atoms are shown using their van der Waals radius (1.66 Å).

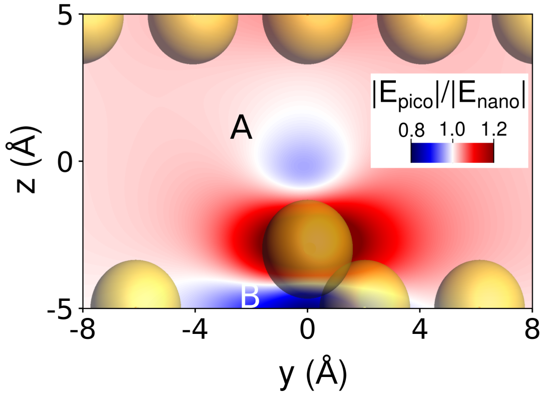

Figure S3: 2D color map of the ratio between picocavity with vacancy and nanocavity electric field enhancement in a zoomed region of the yz plane containing the picocavity adatom. A and B highlight the two depletion zones. Gold atoms are shown using their van der Waals radius (1.66 Å).

S4.2 Picocavities: Adatom

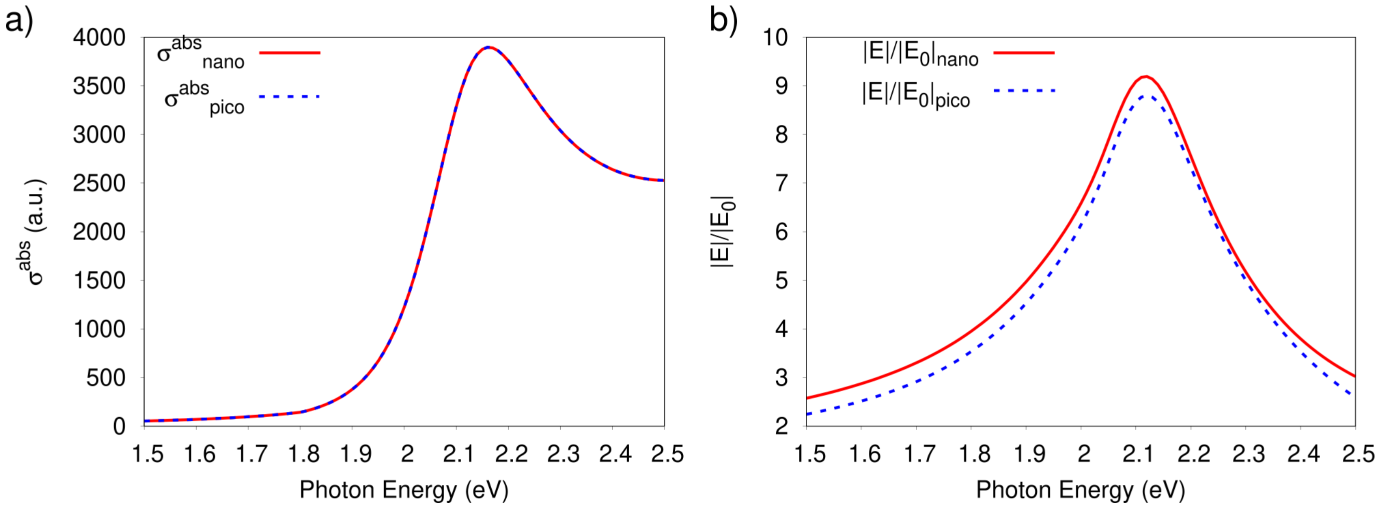

Figure S4: ωFQFμ (a) absorption cross sections σ^{abs} and (b) electric field enhancements ($|\mathbf{E}|/|\mathbf{E}_0|$) calculated for the nanocavity (nano) and picocavity (pico)

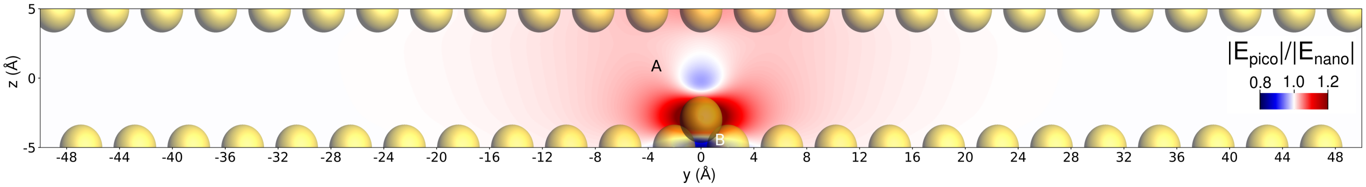

Figure S5: 2D color map of the ratio between picocavity and nanocavity electric field enhancement in a large region of the *yz* plane containing the picocavity adatom. A and B highlight the two depletion zones. Gold atoms are shown using their van der Waals radius (1.66 Å).

References

(1) Giovannini, T.; Rosa, M.; Corni, S.; Cappelli, C. A classical picture of subnanometer junctions: an atomistic Drude approach to nanoplasmonics. *Nanoscale* **2019**, *11*, 6004–6015.

(2) Giovannini, T.; Bonatti, L.; Polini, M.; Cappelli, C. Graphene plasmonics: Fully atomistic approach for realistic structures. *J. Phys. Chem. Lett.* **2020**, *11*, 7595–7602.

(3) Giovannini, T.; Bonatti, L.; Lafiosca, P.; Nicoli, L.; Castagnola, M.; Illobre, P. G.; Corni, S.; Cappelli, C. Do we really need quantum mechanics to describe plasmonic properties of metal nanostructures? *ACS Photonics* **2022**, *9*, 3025–3034.

(4) Giovannini, T.; Puglisi, A.; Ambrosetti, M.; Cappelli, C. Polarizable QM/MM approach with fluctuating charges and fluctuating dipoles: the QM/FQFμ model. *J. Chem. Theory Comput.* **2019**, *15*, 2233–2245.

(5) Haynes, W. *CRC Handbook of Chemistry and Physics*; CRC Press: Boca Raton, 2014.

(6) Bonatti, L.; Gil, G.; Giovannini, T.; Corni, S.; Cappelli, C. Plasmonic Resonances of Metal Nanoparticles: Atomistic vs. Continuum Approaches. *Front. Chem.* **2020**, *8*, 340.

(7) Nicoli, L.; Lafiosca, P.; Grobas Illobre, P.; Bonatti, L.; Giovannini, T.; Cappelli, C. Fully atomistic modeling of plasmonic bimetallic nanoparticles: nanoalloys and core-shell systems. *Front. Photonics* **2023**, *4*, 1199598.

(8) Draine, B. T.; Flatau, P. J. Discrete-dipole approximation for scattering calculations. *JOSA A* **1994**, *11*, 1491–1499.

(9) Corni, S.; Cappelli, C.; Cammi, R.; Tomasi, J. Theoretical approach to the calculation of vibrational Raman spectra in solution within the polarizable continuum model. *J. Phys. Chem. A* **2001**, *105*, 8310–8316.

(10) García de Abajo, F. J.; Howie, A. Retarded field calculation of electron energy loss in inhomogeneous dielectrics. *Phys. Rev. B* **2002**, *65*, 115418.

(11) Trügler, A. *Optical properties of metallic nanoparticles*; Springer, 2011.

(12) Hohenester, U. Quantum corrected model for plasmonic nanoparticles: A boundary element method implementation. *Physical Review B* **2015**, *91*, 205436.

(13) Kunz, K. S.; Luebbers, R. J. *The finite difference time domain method for electromagnetics*; CRC press, 1993.

(14) Lafiosca, P.; Giovannini, T.; Benzi, M.; Cappelli, C. Going beyond the limits of classical atomistic modeling of plasmonic nanostructures. *J. Phys. Chem. C* **2021**, *125*, 23848–23863.
